# A common polymorphism rs3781637 in *MTNR1B *is associated with type 2 diabetes and lipids levels in Han Chinese individuals

**DOI:** 10.1186/1475-2840-10-27

**Published:** 2011-04-06

**Authors:** Yan Ling, Xiaomu Li, Qian Gu, Hongyan Chen, Daru Lu, Xin Gao

**Affiliations:** 1 Department of Endocrinology and Metabolism, Zhongshan Hospital, Fudan University, Shanghai 200032, China; 2 Department of Geriatrics, Zhongshan Hospital, Fudan University, Shanghai 200032, China; 3 The State Key Laboratory of Genetic Engineering and Key Laboratory of Contemporary Anthropology, School of Life Sciences, Fudan University, Shanghai 200433, China

## Abstract

**Background:**

Several studies have shown that common variants in the *MTNR1B *gene were associated with fasting glucose level and type 2 diabetes. The purpose of this study was to examine whether tagging single nucleotide polymorphisms (SNPs) in the *MTNR1B *region were associated with type 2 diabetes and related traits in a Han Chinese population.

**Methods:**

We investigated the association of polymorphisms in the *MTNR1B *gene with type 2 diabetes by employing a case-control study design (1118 cases and 1161 controls). Three tagging SNPs (rs10830963, rs3781637, and rs1562444) with R^2^>0.8 and minor allele frequency>0.05 across the region of the *MTNR1B *gene were studied. Genotyping was performed by matrix-assisted laser desorption/ionization time-of-flight mass spectroscopy using a MassARRAY platform.

**Results:**

The polymorphism rs3781637 was associated with type 2 diabetes adjusted for age, sex and body mass index (BMI) in the additive model and recessive model (OR = 1.22, 95% CI 1.01-1.46, p = 0.038 and OR = 2.81, 95% CI 1.28-6.17, p = 0.01, respectively). In the non-diabetic controls, rs3781637 was nominally associated with plasma triglyceride, total cholesterol and low density lipoprotein cholesterol (LDL-C) levels in the recessive model (p = 0.018, 0.008 and 0.038, respectively). After adjustment for multiple comparisons, the associations of rs3781637 with total cholesterol and LDL-C remained significant in the recessive model (the empirical p = 0.024 and 0.045, respectively), but the association between rs3781637 and triglyceride became non-significant (the empirical p = 0.095). The associations of rs10830963 and rs1562444 with type 2 diabetes and related traits were not significant in the additive, dominant and recessive models.

**Conclusions:**

The rs3781637 A/G polymorphism of the *MTNR1B *gene is associated with type 2 diabetes, plasma, total cholesterol and LDL-C levels in the Han Chinese population.

## Background

Circadian rhythms are closely related to metabolism, and dysregulation of circadian rhythms may increase the risk of diabetes [1]. The *MTNR1B *gene encodes a high affinity receptor for melatonin, a hormone primarily secreted by the pineal gland to regulate circadian rhythm and sleep cycles [2]. Plasma melatonin follows an opposite circadian rhythm to plasma insulin and glucose, rising by night and falling by day [3]. There are favorable evidences that circadian rhythm of melatonin influences insulin secretion and glucose homeostasis via its islet-specific receptor [4]. Consistently, melatonin secretion and circadian rhythm are impaired in type 2 diabetes patients [2]. More importantly, MTNR1B inhibits insulin secretion through its effect on CGMP formation when activated by melatonin [5]. Therefore, the *MTNR1B *gene might be involved in glucose homeostasis and type 2 diabetes.

Several large-scale genome-wide association analyses demonstrated that common variants in or near the *MTNR1B *gene to be robustly associated with fasting glucose level in European populations [6-8], with polymorphism rs10830963 showing the most significant association signal [7]. Several replication studies in European [9-12], Indian [13], Sri Lankan and Japanese populations [14] confirmed that *MTNR1B *rs10830963 contributed to raised fasting glucose level and increased risk of type 2 diabetes.

Several studies investigated the associations of *MTNR1B *rs10830963 with type 2 diabetes and fasting glucose among Han Chinese populations [15-19]. Ling et al. first replicated the association of *MTNR1B *rs10830963 with type 2 diabetes and fasting glucose in a case-control study including 1165 type 2 diabetes cases and 1105 normoglycaemic controls [17]. After that, another four studies reported that *MTNR1B *rs10830963 was associated with type 2 diabetes and/or fasting glucose in Han Chinese populations [15,16,18,19]. Whether other common variants except rs10830963 in the *MTNR1B *gene are associated with type 2 diabetes and fasting glucose in Han Chinese population is unknown.

The aim of this study was to examine whether common polymorphisms in *MTNR1B *were associated with type 2 diabetes and related traits in a Han Chinese population.

## Methods

### Subjects

All participants were of Southern Han Chinese ancestry and resided in the Shanghai metropolitan area. Type 2 diabetic inpatients (n = 1118) were recruited from the Endocrinology and Metabolism Department of Zhongshan Hospital in Shanghai, which is affiliated to Fudan University. All type 2 diabetic patients met the 1999 WHO criteria for diabetes [20], had been diagnosed after the age of 29 years, and were treated with oral hypoglycaemic agents and/or insulin. 1161 unrelated non-diabetic control participants were people undergoing health examinations in Zhongshan Hospital, were older than 40 years, and had a fasting plasma glucose < 5.6 mmol/l.

Written informed consent was obtained from all participants and the study was approved by the ethnic committee of Zhongshan hospital, Fudan University, Shanghai, China.

### Clinical measurements

Both the diabetic patients and the controls were extensively phenotyped for anthropometric and biochemical traits related to glucose metabolism. The phenotypes assessed in our study include height, weight, fasting glucose, total cholesterol, triglyceride, high density lipoprotein cholesterol (HDL-C) and LDL-C. BMI was calculated as weight (kg)/height^2 ^(m^2^).

### Genotyping

We selected tagging single nucleotide polymorphisms (SNPs) with the criteria of R^2^>0.8 and minor allele frequency (MAF)>0.05 across the region of the *MTNR1B *gene (include 3kb upstream and 3kb downstream of the gene) from the HapMap Phase II, using the pairwise tagging model. Pairwise tagging algorithm was developed by Carlson et al., and the detailed description was introduced previously [21]. Three tagging SNPs (rs10830963, rs3781637, and rs1562444) were selected. The genotyping was performed by matrix-assisted laser desorption/ionization time-of-flight mass spectroscopy using a MassARRAY platform (MassARRAY Compact Analyzer, Sequenom, San Diego, CA, USA).

### Statistical analysis

Continuous variables are expressed as means ± standard error of mean (SEM) and median (interquartile range). Comparisons between groups were performed with T testing and χ^2 ^testing for normally distributed continuous and categorical variables, respectively. Deviations from the Hardy-Weinberg equilibrium were assessed by means of χ^2 ^testing. SNPs that were not in the Hardy-Weinberg equilibrium were excluded from further analysis. Additive, dominant and recessive models were assumed in the association analysis. We tested the association of the polymorphisms with type 2 diabetes by using logistic regression. Multivariate linear regression was used to test the association of the polymorphisms with quantitative traits. All models were adjusted for age, sex, and BMI. Permutations (10,000 times) were performed for each trait to assess empirical p values using PLINK[22] in order to adjust the multiple comparisons. Analysis was performed with SPSS software version 17.0.

## Results

### Baseline characteristics

The baseline characteristics of participants in this study are presented in Table 1. In 2279 participants, 1118 were type 2 diabetes patients and 1161 were non-diabetic controls. Diabetic cases were older and had higher BMI, fasting glucose and triglyceride levels, but lower cholesterol concentrations than the non-diabetic controls. There was no significant difference of the distribution of sex between diabetic cases and non-diabetic controls (p = 0.34).

**Table 1:** Baseline characteristics of diabetic cases and non-diabetic controls

Characteristic	Diabetic cases (n = 1118)	Non-diabetic controls (n = 1161)	P value*
Age (years)	60.2 ± 0.37	56.5 ± 0.32	<0.001
Men (%)	44.6	42.7	0.34
BMI (kg/m^2^)	24.4 ± 0.11	23.5 ± 0.09	<0.001
Fasting glucose (mmol/l)	8.17 ± 0.04	4.86 ± 0.01	<0.001
Total cholesterol (mmol/l)	4.37(3.78-5.07)	5.10(4.50-5.80)	<0.001
Triglyceride (mmol/l)	1.47(1.00-2.24)	1.40(1.00-1.90)	<0.001
HDL-C (mmol/l)	1.12(1.00-1.38)	1.30(1.12-1.54)	<0.001
LDL-C (mmol/l)	2.38(1.92-3.01)	3.07(2.50-3.60)	<0.001

Data are expressed as means ± SEM and median (interquartile range)

*p value for comparison between cases and controls

### Associations of *MTNR1B *polymorphisms with type 2 diabetes

Overall, three SNPs (rs10830963, rs3781637, and rs1562444) were selected and genotyped in the present study. The call rates of rs10830963, rs3781637, and rs1562444 were 98.0%, 98.5% and 98.0%, respectively. The concordant rates of all SNPs based on 120 duplicates were 100%.

rs10830963, rs3781637, and rs1562444 were in Hardy-Weinberg equilibrium in the study population (Table 2). The polymorphism rs3781637 was nominally associated with type 2 diabetes adjusted for age, sex and BMI in the additive and recessive models (p = 0.038 and 0.01, respectively) (Table 3). After adjustment for multiple comparisons, the association of rs3781637 with type 2 diabetes remained significant in the additive and recessive models (the empirical p = 0.050 and 0.018, respectively). The carriers of GG genotype was associated with higher odds of type 2 diabetes compared with the carriers of AA and AG genotypes adjusted for age, sex and BMI (OR = 2.81, 95% CI 1.28-6.17, p = 0.01) (Table 3). The associations of rs10830963 and rs1562444 with type 2 diabetes were not significant in the additive, dominant and recessive models (Table 3).

**Table 2:** Characteristics of SNPs genotyped in *MTNR1B*

	SNP identification	Chromosome Position	Relation to the gene	Major allele	Minor allele	MAF	HWE P value
1	rs10830963	92348358	Intron	C	G	0.40	0.13
2	rs3781637	92353418	Intron	A	G	0.14	0.13
3	rs1562444	92355497	3'UTR	A	G	0.31	0.14

**Table 3:** Genotypic and allelic distribution of *MTNR1B *polymorphisms and association with type 2 diabetes

SNP	Alleles Major/minor	MAF	Genotype Frequency T2DM cases/controls	OR_add_ (95%CI) ^a^	P value*	OR_dom_ (95%CI)^b^	P value*	OR_rec_ (95%CI) ^c^	P value*
rs10830963	C/G	0.40	0.361/0.481/0.159	0.92	0.22	0.85	0.08	1.01	0.96
			0.348/0.508/0.144	(0.81-1.05)		(0.70-1.02)		(0.78-1.30)	
rs3781637	A/G	0.14	0.717/0.260/0.022	1.22	0.038	1.17	0.13	2.81	0.01
			0.736/0.252/0.012	(1.01-1.46)		(0.96-1.43)		(1.28-6.17)	
rs1562444	A/G	0.31	0.472/0.432/0.096	1.06	0.42	1.02	0.83	1.27	0.14
			0.457/0.456/0.088	(0.92-1.22)		(0.85-1.22)		(0.92-1.74)	

^a ^Calculated using logistic regression, assuming an additive model adjusted for age, sex and BMI

^b ^Calculated using logistic regression, assuming a dominant model adjusted for age, sex and BMI

^c ^Calculated using logistic regression, assuming a recessive model adjusted for age, sex and BMI

* P values shown are not corrected for multiple comparisons

MAF: minor allele frequency in control samples; T2DM: type 2 diabetes.

### Associations of *MTNR1B *polymorphisms with quantitative traits in non-diabetic controls

**Figure 1:**
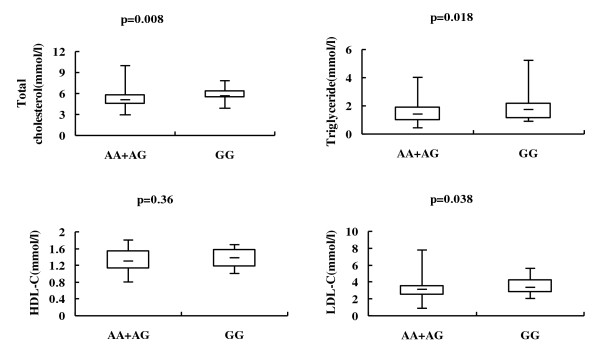
**Associations of rs3781637 with lipid levels**. The upper, middle and lower lines represent the maximum, the median and the minimum, respectively. The bottom and the top of the box represent the 25^th ^and 75^th ^percentiles, respectively. All p values shown are calculated using multivariate linear regression, assuming a recessive model adjusted for age, sex and BMI, and are not corrected for multiple comparisons.

In the non-diabetic controls, the polymorphism rs3781637 was nominally associated with plasma triglyceride, total cholesterol and LDL-C levels adjusted for age, sex and BMI in the recessive model (p = 0.018, 0.008 and 0.038, respectively) (Table 4, Figure 1). The p values of the associations of rs3781637 with plasma triglyceride, total cholesterol and LDL-C levels were 0.011, 0.006 and 0.036 after additional adjustment for fasting glucose. After adjustment for multiple comparisons, the associations of rs3781637 with total cholesterol and LDL-C remained significant in the recessive model (the empirical p = 0.024 and 0.045, respectively), but the association between rs3781637 and triglyceride became non-significant (the empirical p = 0.095). The carriers of GG genotype had a significantly higher plasma total cholesterol and LDL-C levels compared with the carriers of AA and AG genotypes adjusted for age, sex and BMI, and the effect sizes for the GG genotype were 0.841 and 0.585 mmol/l, respectively (Table 4). The polymorphism rs3781637 was not associated with fasting plasma glucose and HDL-C levels in the additive, dominant and recessive models (Table 4). The associations of rs10830963 and rs1562444 with fasting plasma glucose, plasma triglyceride, total cholesterol, LDL-C and HDL-C levels were not significant in all three models (Table 4).

**Table 4:** Association between *MTNR1B *polymorphisms and quantitative traits in non-diabetic controls

rs10830963									
**Genotype**	**CC**	**CG**	**GG**	**β_add_ ± SEM ^a^**	P_add_^a^*	**β_dom_ ± SEM ^b^**	P_dom_^b^*	**β_rec_ ± SEM ^c^**	P_rec_^c^*
n	396	578	164						
Fasting glucose (mmol/l)	4.86 ± 0.02	4.87 ± 0.02	4.84 ± 0.03	-0.009 ± 0.017	0.59	0.0004 ± 0.024	0.99	-0.033 ± 0.032	0.30
Triglyceride (mmol/l)	1.40(1.10-1.90)	1.40(1.00-1.90)	1.40(1.10-1.80)	-0.062 ± 0.049	0.21	-0.079 ± 0.070	0.25	-0.080 ± 0.094	0.40
Total cholesterol (mmol/l)	5.20(4.50-5.90)	5.10(4.50-5.73)	5.10(4.50-5.80)	-0.015 ± 0.045	0.74	-0.042 ± 0.063	0.50	0.022 ± 0.084	0.79
LDL-C (mmol/l)	3.10(2.54-3.64)	3.09(2.50-3.57)	3.09(2.57-3.67)	-0.002 ± 0.039	0.97	-0.020 ± 0.055	0.72	0.030 ± 0.075	0.69
HDL-C (mmol/l)	1.30(1.10-1.60)	1.40(1.13-1.53)	1.40(1.14-1.50)	-0.003 ± 0.014	0.84	-0.011 ± 0.019	0.57	0.010 ± 0.026	0.70

**rs3781637**									

**Genotype**	**AA**	**AG**	**GG**	**β_add_ ± SEM ^a^**	P_add_^a^*	**β_dom_ ± SEM ^b^**	P_dom_^b^*	**β_rec_ ± SEM ^c^**	P_rec_^c^*
n	841	288	14						
Fasting glucose (mmol/l)	4.87 ± 0.01	4.84 ± 0.02	4.78 ± 0.10	-0.037 ± 0.025	0.14	-0.035 ± 0.026	0.18	-0.129 ± 0.124	0.30
Triglyceride (mmol/l)	1.40(1.00-1.90)	1.40(1.00-1.90)	1.70(1.13-2.18)	0.032 ± 0.071	0.65	0.002 ± 0.075	0.98	0.742 ± 0.314	0.018
Total cholesterol (mmol/l)	5.10(4.50-5.80)	5.10(4.40-5.80)	5.60(5.40-6.38)	-0.022 ± 0.065	0.74	-0.062 ± 0.069	0.367	0.841 ± 0.316	0.008
LDL-C (mmol/l)	3.07(2.55-3.62)	3.02(2.50-3.56)	3.36(2.81-4.26)	-0.021 ± 0.057	0.71	-0.050 ± 0.060	0.40	0.585 ± 0.281	0.038
HDL-C (mmol/l)	1.30(1.13-1.53)	1.30(1.11-1.60)	1.38(1.17-1.59)	-0.001 ± 0.020	0.95	0.003 ± 0.021	0.89	-0.086 ± 0.095	0.36

**rs1562444**									

**Genotype**	**AA**	**AG**	**GG**	**β_add_ ± SEM ^a^**	P_add_^a^*	**β_dom_ ± SEM ^b^**	P_dom_^b^*	**β_rec_ ± SEM ^c^**	P_rec_^c^*
n	520	518	100						
Fasting glucose (mmol/l)	4.86 ± 0.02	4.86 ± 0.02	4.87 ± 0.04	0.005 ± 0.018	0.79	0.005 ± 0.023	0.83	0.009 ± 0.042	0.83
Triglyceride (mmol/l)	1.40(1.00-1.90)	1.40(1.00-1.90)	1.40(0.90-1.90)	0.009 ± 0.047	0.85	0.046 ± 0.060	0.44	-0.108 ± 0.109	0.32
Total cholesterol (mmol/l)	5.10(4.50-5.80)	5.10(4.50-5.80)	5.10(4.40-5.90)	-0.004 ± 0.047	0.94	-0.019 ± 0.060	0.75	0.046 ± 0.110	0.67
LDL-C (mmol/l)	3.11(2.57-3.64)	3.03(2.50-3.60)	3.00(2.59-3.58)	-0.022 ± 0.042	0.60	-0.041 ± 0.053	0.44	0.020 ± 0.097	0.84
HDL-C (mmol/l)	1.30(1.13-1.51)	1.30(1.11-1.54)	1.36(1.16-1.65)	0.010 ± 0.014	0.51	0.001 ± 0.018	0.97	0.049 ± 0.033	0.14

Data are expressed as means ± SEM and median (interquartile range)

^a ^Calculated using multivariate linear regression, assuming an additive model adjusted for age, sex and BMI

^b ^Calculated using multivariate linear regression, assuming a dominant model adjusted for age, sex and BMI

^c ^Calculated using multivariate linear regression, assuming a recessive model adjusted for age, sex and BMI

* P values shown are not corrected for multiple comparison

## Discussion

Here we reported that a common genetic variant rs3781637 in *MTNR1B *was associated with type 2 diabetes in a Han Chinese population. The association of rs3781637 with type 2 diabetes was found to be significant in the additive and recessive models. In the recessive model, the carriers of GG genotype were associated to higher odds of type 2 diabetes compared with the carriers of AA and AG genotypes (OR = 2.81). In addition, rs3781637 showed nominal association with plasma triglyceride, total cholesterol and LDL-C levels in the recessive model in non-diabetic controls, and the associations of rs3781637 with total cholesterol and LDL-C levels remained significant after adjustment for multiple comparisons. The carriers of GG genotype were associated to a higher total cholesterol and LDL-C levels compared with the carriers of AA and AG genotypes. In addition, the associations of rs3781637 with lipid levels were independent of glucose metabolism. The association between *MTNR1B *variants and lipid profile was not reported before, and the mechanism underlying this association remains to be determined. However, many evidences showed that melatonin played an important role in the lipid metabolism. Animal studies showed that melatonin treatment significantly improved dyslipidemia in diabetic rats, with reductions in triglyceride and LDL-C levels [23,24]. The administration of melatonin and zinc was found to improve the lipid profile in type 2 diabetic patients [25]. A novel melatonin agonist, NEU-P11, decreased total cholesterol and triglyceride levels, while increased HDL-C level in obese rats [26]. Therefore, the *MTNR1B *gene which encoded a high affinity receptor for melatonin may be involved in the lipid metabolism.

We failed to replicate the association of rs10830963 with type 2 diabetes or fasting plasma glucose in our Han Chinese population. However, another three case-control studies in Shanghai Han Chinese populations have reported the significant association of rs10830963 with type 2 diabetes and/or fasting plasma glucose [16-18]. Inconsistent results are often seen between different genetic association studies. Here, population heterogeneity and different environmental factors are unlikely responsible for the inconsistency between studies. Because all study populations had Han ancestry, were from Shanghai metropolitan area, and had similar lifestyle. In addition, the allele frequency of rs10830963 in our study is similar to those in other studies, and the call rate and concordant rate of rs10830963 genotyping in our study are 98.0% and 100%, respectively. Genotyping error could not be the reason for the discrepancy between our study and other studies. Therefore, more replication studies of *MTNR1B *polymorphisms in Han Chinese and meta-analysis are needed in the future.

Recently, *MTNR1B *polymorphism rs10830963 was shown to be associated with fasting glucose and homeostasis model assessment of βcell function (HOMA-β) in Caucasian overweight and obese children and adolescents[27], which suggests that the effects of *MTNR1B *polymorphisms on glucose metabolism were not limited to adult populations.

As diabetes is one of the risk equivalents of coronary heart disease (CAD), and the *MTNR1B *polymorphism is associated with type 2 diabetes and lipid levels in the present study, do they also contribute to the development of CAD? Until now, there is no report about the association between *MTNR1B *polymorphisms and CAD. Some other diabetes-related gene polymorphisms such as *KCNQ1 *gene polymorphisms [28-30]and uncoupling protein 2 (*UCP2*) gene polymorphisms [31] have been proved to be associated with CAD or the risk factors of CAD. Recently, a polymorphism rs1333049 on chromosome 9p21.3 was found to be associated with CAD and coronary plaque progression in non-diabetic but not in type 2 diabetic patients[32], implying that it affects the development of CAD through a novel biological pathway other than interaction with diabetes or glucose metabolism. Whether polymorphisms in *MTNR1B *contribute to the susceptibility of CAD, and whether they contribute to diabetes and CAD through a common pathway should be investigated in the future study.

There is strong epidemiologic, clinical and phenotypic overlap between type 2 diabetes and some insulin resistance-related conditions, such as polycystic ovary syndrome (PCOS) [33]. One study investigated the relationship between *MTNR1B *polymorphisms and PCOS among Chinese women, and found that rs10830963 in *MTNR1B *is not only associated with susceptibility to PCOS, but also contribute to the PCOS phenotype[34]. However, the authors didn't provide any information about the difference of plasma glucose between the PCOS patients and control subjects. It is possible that the association of *MTNR1B *polymorphism with PCOS was confounded by the plasma glucose level which tends to be higher in PCOS patients. Further studies are needed to determine whether the association of *MTNR1B *polymorphisms with PCOS is mediated through its effect on glucose metabolism or is independent of glucose metabolism.

Recent studies also found that *MTNR1B *polymorphisms are associated with adolescent idiopathic scoliosis (AIS) [35,36], a complex deformity of the spine that most commonly occurs in girls at the peripubertal period between 10 and 16 years of age. This finding is not so surprising because of the fact that melatonin can induce osteoblast differentiation from human mesenchymal stem cells and plays an important role in osteogenesis[37,38]. Studies with animal models, such as pinealectomized chickens[39], pinealectomized bipedal rats[40], and bipedal C57BL/6J mice with genetically low circulating melatonin levels[41], suggested that melatonin deficiency could have a significant role in the development of AIS. Besides melatonin, hormones like leptin[42], adiponectin[43], glucagon-like peptide-1[44] and 1, 25-dihydroxy-vitamin D[45] all effect glucose metabolism and bone metabolism simultaneously.

The associations of *MTNR1B *rs3781637 with type 2 diabetes and lipids levels found in our study were not reported in the previous studies, and the function of rs3781637 is unknown. The limitation of our study is that we didn't do functional study of rs3781637. It is located in the intron of *MTNR1B*. It may be associated with alternative splicing of mRNA or the binding of transcription factor and affects the expression level of protein. Future functional study is needed to demonstrate this. There is also no evidence that rs3781637 is linked with any functional variant now, but future fine mapping and resequencing of the *MTNR1B *gene may detect such functional variants.

## Conclusions

Our study demonstrated that *MTNR1B *rs3781637 A/G polymorphism is associated with type 2 diabetes, total cholesterol and LDL-C levels in the Han Chinese population. These observations suggest that *MTNR1B *variants may be involved in both glucose and lipid metabolism, which warrants further studies using larger, independent cohorts and a population-based approach.

## Abbreviations

SNP: single nucleotide polymorphism; MAF: minor allele frequency; LD: linkage disequilibrium; SEM: standard error of mean; BMI: body mass index; CI: confidence interval; OR: odds ratio; HDL-C: high density lipoprotein cholesterol; LDL-C: low density lipoprotein cholesterol; T2DM: type 2 diabetes; HWE: Hardy-Weinberg equilibrium; HOMA-β: homeostasis model assessment of βcell function; CAD: coronary heart disease; UCP2: uncoupling protein 2; PCOS: polycystic ovary syndrome; AIS: adolescent idiopathic scoliosis.

## Competing interests

The authors declare that they have no competing interests.

## Authors' contributions

YL participated in the design of the study, carried out the SNP genotyping and the statistical analysis of the genotype data, and drafted the manuscript. XG contributed to the design and coordination of the study, to the statistical analysis, interpreted the findings and drafted the manuscript. XL, QG, HC and DL participated in the design of the study and the SNP genotyping. All authors read and approved the final manuscript.
